# Efficient Data Transfer Rate and Speed of Secured Ethernet Interface System

**DOI:** 10.1155/2016/9458627

**Published:** 2016-12-27

**Authors:** Shaila Ghanti, G. M. Naik

**Affiliations:** Department of Electronics, Goa University, Goa, India

## Abstract

Embedded systems are extensively used in home automation systems, small office systems, vehicle communication systems, and health service systems. The services provided by these systems are available on the Internet and these services need to be protected. Security features like IP filtering, UDP protection, or TCP protection need to be implemented depending on the specific application used by the device. Every device on the Internet must have network interface. This paper proposes the design of the embedded Secured Ethernet Interface System to protect the service available on the Internet against the SYN flood attack. In this experimental study, Secured Ethernet Interface System is customized to protect the web service against the SYN flood attack. Secured Ethernet Interface System is implemented on ALTERA Stratix IV FPGA as a system on chip and uses the modified SYN flood attack protection method. The experimental results using Secured Ethernet Interface System indicate increase in number of genuine clients getting service from the server, considerable improvement in the data transfer rate, and better response time during the SYN flood attack.

## 1. Introduction

Evans [1] has suggested that Internet evolution in future consists of large number of smart devices used in home automation, vehicular communications, and Internet of things. These devices used in providing many services are prone to attack on the Internet. These services need to be secured and the type of security depends on the service that is provided. Depending on the specific application provided by the system, one of the protection methods like IP filtering, TCP protection, UDP protection, ICMP protection, and so on could be used. Every device uses network interface to communicate on the Internet and the security of these devices is vital; hence there is a need to study the effect of security systems with the network interface.

In order to provide security to different online automation systems that monitor and control physical devices, there is a need to understand the way these systems are designed. Depending on the type of application, different ways of implementing these automation systems are evolving. Web enabled communication devices are used for monitoring and controlling physical objects over the Internet. Most of the automation systems use Wi-Fi rather than wired cables. If the device needs to move from one place to another, wireless is a good choice, and if fixed devices like TV, motors, and so on are to be controlled remotely then the best option would be to use a wired cable. Wired Ethernet offers advantages of faster speed, lower latency, and no wireless inference as indicated in the article [2]. According to Hahm et al. [3], Al-Fuqaha et al. [4] embedded systems like Arduino, Raspberry PI, Beagle Bone Black, Econotag, and so on are generally used in designing the IOT devices. Raspberry PI is used by Vujović and Maksimović [5, 6] as web sensor node, which can be connected to the Internet via an Ethernet/LAN cable or USB dongle. Raspberry Pi is used to serve static websites and is implemented to communicate with sensor units via GPIO (12C) interface. The web enabled automation system uses the web server that can be part of sensor device or default gateway. Remote clients through the Internet connect to the web sensor node and using web application the devices are monitored and controlled. These servers used in an automation system need to be secured.

This paper proposes the design of the embedded* Secured Ethernet Interface System*.* Secured Ethernet Interface System* can be used to protect the Internet services provided by home automation systems, small office system, vehicle system, health system, and so on.* Secured Ethernet Interface System* can be customized to provide the security features like IP filtering, UDP protection, or TCP protection, and so on, depending on the specific application. Most of the services on the Internet are web based services. However, access to these services can be blocked by Denial of Service (DoS)/Distributed Denial of Service (DDoS) attacks [7]. Hence in this experimental study the* Secured Ethernet Interface System* is customized to protect the web service against the SYN flood attack which is a type of DDoS attack. To study the effect of Triple-Speed Ethernet in a security system the proposed system uses NIOS II softcore processor and Triple-Speed Ethernet IP core. The proposed* Secured Ethernet Interface System* is implemented on ALTERA Stratix IV FPGA as a system on chip and uses the modified SYN flood attack protection method. Experiments were conducted to test the performance of* Secured Ethernet Interface System* and it was found that there was increase in number of genuine clients getting the service from the server, considerable improvement in the data transfer rate, and enhanced response time.

## 2. SYN Flood Attack Overview and Literature Survey

TCP connection needs to be set up between the server and the client on the Internet to access the TCP service provided by the server. TCP connection setup involves three-way hand shake between the client and the server. First the SYN request is sent from the client to the server to set up connection; the server sets up the half-open connection and sends back the SYN-ACK to the client. The client in response to the SYN-ACK sends back ACK packets to the server and the connection is set up between the client and the server.

SYN flood attack is a type of DDoS attack that sends large number of spoofed SYN attack packets to the server where in the three-way handshake of TCP connection remains incomplete. This consumes large memory of the server that leads to a resource crunch and restricts the genuine clients access to the server [8]

The spoofed SYN flood attack packets are similar to the genuine client requests and, hence, it is very difficult to detect and protect the SYN flood attack [8]. SYN flood attack detection methods are basically classified based on router data structure, statistical analysis of packet flow, and artificial intelligence [9]. On comparing the performances of the SYN flood attack defense mechanisms it is found that the router based SYN flood defense mechanism results were better [10]. Therefore, victim side defending mechanism based on router is used in our proposal.

There are many methods available in the literature to prevent the SYN flood attack like ingress filtering [11], SYN cache [12], SYN cookies [13], use of matching SYN and SYN/ACK address and ARP protocol [14], use of all valid IP address database to filter the attack [15], software defined network solutions [16], and so on.

The following papers have reported the use of Field Programmable Gate Arrays (FPGA) for network applications and securities like the implementation of web server on FPGA [17], implementation of security of web server using FPGA [18], server protection for an application using FPGA [19], design of firewall to filter the packets based on IP address, MAC address, port number using NIOS processor and customized hardware (like CAM) [20], and design and implementation for maintaining a centralized repository unit [21].

The design of some of the automation systems is mentioned below. Wong and Kapila [22] have implemented the automation system wherein the remote clients can control DC motor. Embedded Tiny Internet Interface microcontroller is configured as web server. Remote clients connect to this automation system web server and control the DC motor. Web based home automation system to control the electrical appliances and monitor the electrical consumption is designed by Putra et al. [23]. In this the hardware Raspberry Pi, Arduino board, and CT current sensors are used with Wi-Fi or Ethernet connection. Can Filibeli et al. [24] have implemented the web based home automation system for controlling the home appliances

These automation systems can be easily implemented on a FPGA as a single system on chip or network on chip. The challenges of designing IOT devices include power efficiency and interoperability can be solved with the use of FPGA which offers low cost, small size, and very low power solutions [25].

## 3. Proposed Design of Secured Ethernet Interface System

To study the effect of Triple-Speed Ethernet in a security system the proposed system uses Triple-Speed Ethernet and NIOS II softcore processor to process the security method. The proposed design of* Secured Ethernet Interface System* involves two steps: (i) method that is used to protect the system and (ii) design and implementation of the* Secured Ethernet Interface System* hardware using the FPGA.

**Figure 1:**
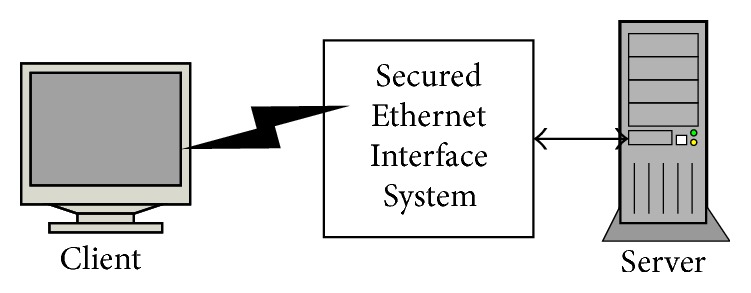
*Secured Ethernet Interface System* between the server and client.

The* Secured Ethernet Interface System* is to be connected between the clients and the server as shown in Figure 1.

### 3.1. Method Used to Protect the System

**Figure 2:**
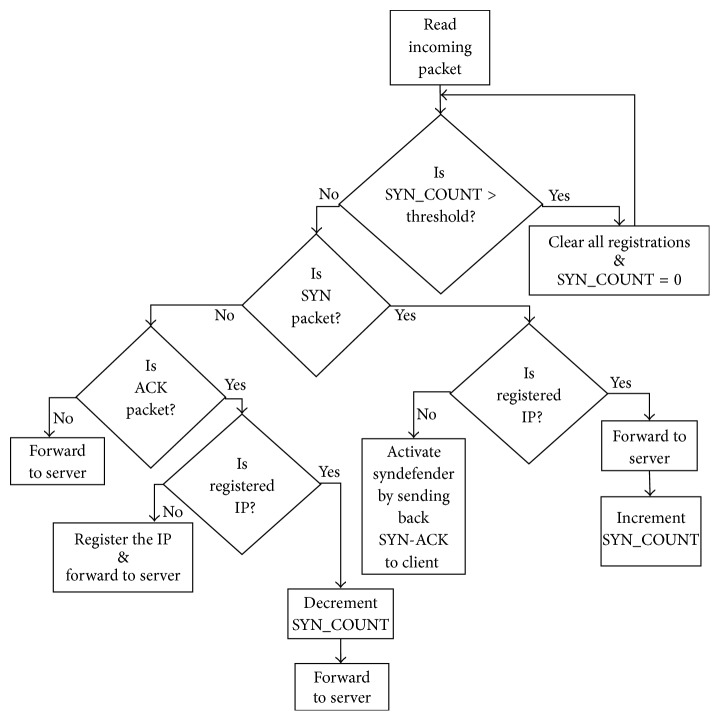
SYN flood attack protection method used by* Secured Ethernet Interface System*.

The proposed modified SYN flood attack protection method is shown in Figure 2 and is based on [26–29]. In order to protect the server from SYN flood attack there is a need to identify the SYN requests coming from genuine client or attacker so that the attacker's requests can be blocked. It is very difficult to identify attacker's requests as they use the spoofed IP addresses. In this method spoofed IP attack packets can also be identified and the attack on server is mitigated.* Secured Ethernet Interface System* uses this method to protect the server.

In this study, we have classified all the incoming client IP addresses into 3 categories:

- Registered IP address: the client IP address that had a successful connection with the server and is registered in the registry of the* Secured Ethernet Interface System* is treated as genuine client IP address
- Nonregistered IP address: the client IP address that is not present in the registry of the* Secured Ethernet Interface System* is treated as new client IP address
- Spoofed IP address: the IP address present in the attack packet from an attacker is a spoofed IP address. The spoofed IP address can be of two types. First is the spoofed IP address that is not present in the registry of the* Secured Ethernet Interface System* and is treated as new client IP address. Second is the spoofed IP address that is present in the registry of the* Secured Ethernet Interface System* and is treated as spoofed IP address.

Initially the registry is empty and the SYN_COUNT is zero. When a client sends the SYN request to the protected server, the client IP address is not found in the registry and is a nonregistered IP address. As shown in Figure 2 the client request with the nonregistered IP address is not forwarded to the server; instead the protection system sends back SYN-ACK to the client [26]. If the client is a genuine then the corresponding ACK is received and the* Secured Ethernet Interface System* adds the client IP address in the registry by setting the connection to the server.

When a client request with the registered IP address is received by the* Secured Ethernet Interface System* it forwards the request to the server and the SYN_COUNT is incremented. The server replies with SYN-ACK and since the request is from genuine client the corresponding ACK is received to set up the connection and the SYN_COUNT is decremented. Hence the SYN_COUNT value does not change due to the request received from the registered genuine client. Thus, the registered and nonregistered genuine client requests do not change the SYN_COUNT value.

The requests with the spoofed IP addresses generated by attacker are handled by the* Secured Ethernet Interface System* in the following manner.

SYN requests with nonregistered IP addresses (spoofed) are not forwarded to the server; instead the* Secured Ethernet Interface System* sends back SYN-ACK. The corresponding ACK will not be received by the server as it is from attacker. Thus, the spoofed SYN flood attack requests with nonregistered IP addresses do not affect the server and such attacks are immediately blocked.

The important feature of this algorithm is to block the registered spoofed SYN flood attack. In case an attacker sends SYN request with the spoofed registered IP address, then the request is forwarded to the server since the IP address is already present in the registry. For every forwarded request the SYN_COUNT is incremented and half-open connection is set up on the server. As the request is from attacker with the spoofed IP address the corresponding ACK will not be received. It is possible that during SYN flood attack large number of spoofed requests are forwarded to the server and all the server resources could be utilized in setting half-open connections. This could lead to denial of service to the genuine clients. In order to protect the server from registered spoofed SYN flood attack this method provides an innovative solution. For every incoming packet the Secured Ethernet Interface System keeps on checking the SYN_COUNT value. If the SYN_COUNT value is greater than the threshold value, then all the registered IP addresses from the registry are cleared and set the SYN_COUNT = 0. Once the registry is cleared further spoofed SYN attack requests are blocked and not forwarded to the server. However, further requests from the genuine clients are treated as new requests and once the corresponding ACKs are received then these new IP addresses will be added to the registry.

Thus, this method protects the server from SYN flood attack by blocking the spoofed requests and the genuine clients' access to the service is not denied.

Method based on [29] generates the RST packets to clear the half-open connections set up on the server once the attack is detected. Also, an IP registry was updated and deleted for every incoming packet depending on if the corresponding ACK is received or not. In contrast to the method [29], the proposed modified algorithm does not make use of RST packets and also in this method the IP addresses are added in the registry only if the request is from a new genuine client. Due to that, this proposed method results in improved speed of processing and faster response during the attack.

The main hurdle in this proposed method is maintaining huge registry of IP addresses and comparing an IP address of the incoming SYN request with the registry. This problem can be overcome using the space efficient data structure bloom filter as indicated by [30]. The bloom filter uses much less memory space; it is much easier to implement especially in FPGA and much faster to process.

### 3.2. Design of Implementation of Secured Ethernet Interface System Hardware Using FPGA

**Figure 3:**
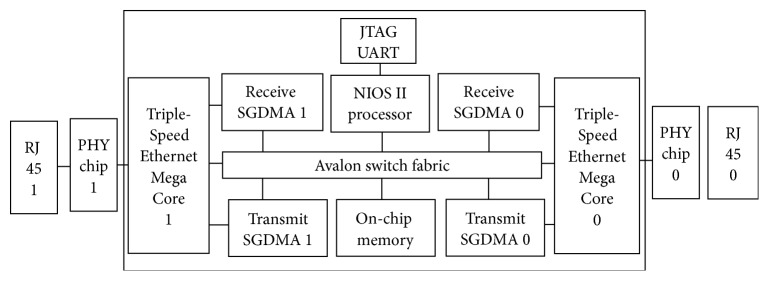
Block diagram of basic hardware* Secured Ethernet Interface System* using FPGA.

FPGA is a reprogrammable device that provides high performance. The FPGA based* Secured Ethernet Interface System* is designed using IP cores like NIOS II/s standard processor, Triple-Speed Ethernet, Transmit and Receive Scatter Gather DMA Controller (SGDMA), on-chip memory, JTAG UART, PLL, and 88E1111 Ethernet PHY chip [31–34] and the layout is as shown in Figure 3. The clock frequency for this system is set to 100 MHz. NIOS softcore processor is used as a processing unit. Hardware Description Language (HDL) is used to develop NIOS. NIOS II is a 32-bit processor that uses RISC architecture and can be programmed using c/c++ high level programming language. The NIOS II processor is used for running the application program to process the packet headers to identify the spoofed attack packets and then block the attack so that the effect of attack is mitigated. The Triple-Speed Ethernet is a soft Intellectual Property (IP) core that provides Media Access Control features. The JTAG UART component is used to communicate between the host computer and the processor. The Transmit and Receive SGDMA are used for transmitting and receiving functions of the core. SGDMA controller is used to transfer data from streaming interface to memory-mapped interface and memory-mapped interface to streaming interface. The on-chip memory is used to store the programs and packets received from the TSE core as well as SGDMA descriptors. The Phase Locked Loop (PLL) module will take 50 MHz input clock and generate the required clock output of 100 MHz and 125 MHz. The physical layer functionalities are provided by 88E1111 Ethernet PHY chip, which is supported by the Altera Mega Core Triple-Speed Ethernet, a softcore IP that provides functionalities performed by the MAC layer.

Basic FPGA hardware* Secured Ethernet Interface System* as shown in Figure 3 is implemented on Startix IV GX FPGA [35]. Quartus 11.1 builder is used for designing, compiling, and executing and testing the NIOS based programs. QSYS tool [36] is a tool used for developing system on chip hardware. Quartus 11.1 and Qsys tool are used to add the abovesaid components, generate Verilog code, and perform the pin assignments to the board, and then the project is compiled. After compiling, the FPGA is programmed and configured to implement the designed circuit.

An application program is executed on the above described hardware to protect the server from SYN flood attack. An application program begins with the initialization of SGDMA, Triple-Speed Ethernet, and PHY device. Then the SGDMA transmit and receive device is created, descriptors in the descriptor memory are allocated, all the SGDMA devices are opened, and interrupts for SGDMA devices are to be set. Initialize both Triple-Speed Ethernets with the base address, assign the required MAC address in a register, specify the address of the required PHY device to be accessed through MDIO interface, set the PCS to operate at SGMII mode, and enable SGMII autonegotiation. Set PHY address for accessing the PHY chip for Ethernet port 2 and another for port 1, enable automatic crossover for all modes of the PHY, and software-reset the PHY chip and wait. Then we enable read and write transfers and CRC forwarding and create both SGDMA receive descriptors for nonblocking transfer [34].

*ISR Routine*. For every incoming packet the interrupt is generated so that ISR can be used to read the incoming packet and process the packet according to the modified SYN flood attack protection method as shown in Figure 2. The logic utilization of the* Secured Ethernet Interface System* implemented on FPGA is only 8%.

## 4. The Experimental Setup

To study the effectiveness of* Secured Ethernet Interface System*, two setups were used: protected server and unprotected server.

**Figure 4:**
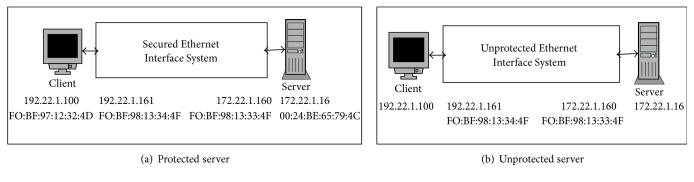
Experimental setup.

*Protected Server*. The FPGA* Secured Ethernet Interface System* designed as described earlier in Sections 3.1 and 3.2 is connected between the client and the server as shown in Figure 4(a) and the server is said to be a protected server.

*Unprotected Server*. The FPGA based Unprotected Ethernet Interface System uses the hardware as shown in Figure 3. But the ISR routine is changed wherein every incoming packet is forwarded to the server and vice versa. The Unprotected Ethernet Interface System acts as a simple FPGA router. The server is said to be unprotected server when this Unprotected Ethernet Interface System is connected between the client and server as shown in Figure 4(b).

For the protected server the experimental setup is shown in Figure 4(a) and for the unprotected server experimental setup is shown in Figure 4(b). In both the experiments different number of genuine client requests are sent to the server in the presence of attacks and the following parameters are measured:

- Total number of half-open connections set up on the server
- Total number of connections set up on the server
- Time taken by the server to respond
- Data transfer rate

To generate the genuine client requests the* ab* tool is used and Ostinato packet generator tool is used to generate SYN flood attack packets [37–39].

## 5. Results and Discussion

### 5.1. Results

**Figure 5:**
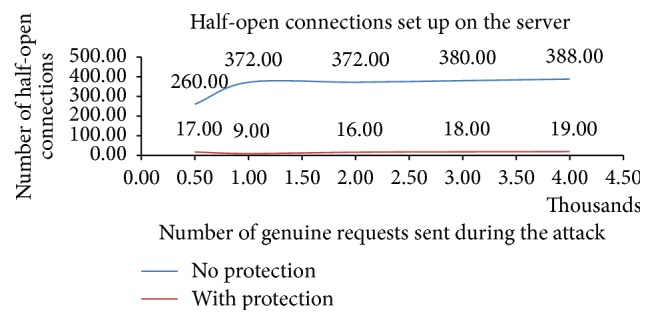
Half-open connections set up on the protected and unprotected server.

**Figure 6:**
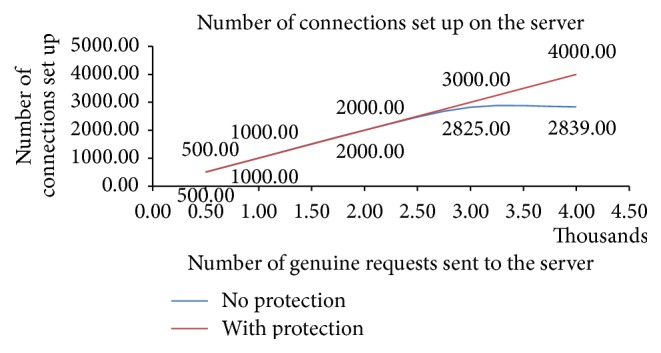
Response of server during attack with and without FPGA SYN flood attack security system.

**Figure 7:**
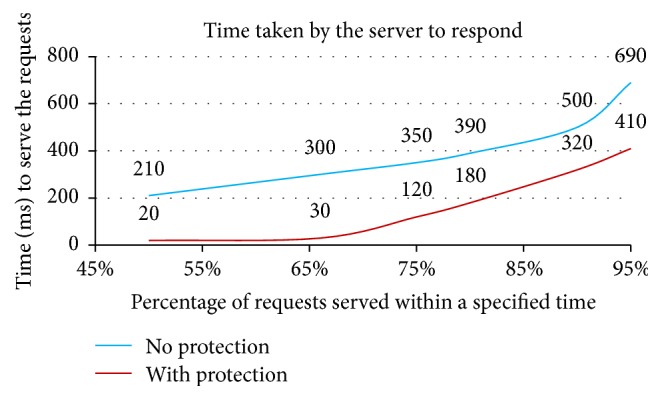
Percentage of requests served with and without the* Secured Ethernet Interface System*.

The results of the experiments indicated in Figures 5, 6, and 7 and Table 1 are discussed here:

1. Figure 5 depicts that the number of half-open connections set up on the protected server is much less as compared to the server that is not protected. The* Secured Ethernet Interface System* detects and blocks the attack. Thus attack packets are not forwarded to the server and subsequently the number of half-open connections set up on the server is comparatively much less. This results in minimum wastage of resources and allows large number of clients to access the service from the server. Thus, the effect of attack on the server is mitigated.
2. The number of connections set up on the server represents the number of clients that have got the service from the server. The number of connections set up on the server is more when the server is protected as compared to when the server is not protected as shown in Figure 6. This indicates that in spite of the attack large number of clients can access the protected server; otherwise these clients would have been denied the access to the server.
3. 500 genuine client requests are sent to the protected and unprotected server with the attack and then the percentage of requests served within specified time is measured as shown in Figure 7. Response time of server (95% of requests served without protection) is 690 ms. Response time of server (95% of requests served with protection) is 410 ms. Time taken by the clients to get service from protected server is much less as compared to the unprotected server.
4. Table 1 shows the transfer rate between the client and the server and the time taken to test the 500 genuine client requests during the attack when the server is protected and not protected. This table indicates that protected server data transfer rate is improved and less time is taken to process the client requests, as compared to unprotected server.

### 5.2. Discussion

Embedded systems are extensively used in automation systems that monitor and control the physical objects like light, heater, DC motor, and so on. These online automation systems are activated by user remotely. These automation systems make use of different technologies, like wired, wireless, Zigbee, TCP/IP, and so on. Most of the online automation systems are web based. Automation systems use cloud services or the services provided on the system depending on the need of the application. If the application needs to store a lot of data then the cloud services can be used; otherwise the web server can be configured on the embedded system used in the automation systems. These web services provided by the automation system can be accessed by client remotely and can activate the physical objects. The majority of the web based automation systems are designed without any concern to their security. There are different types of securities like encryption, filtering, TCP attack protection, and UDP attack protection that needs to be provided. The proposed Secured Ethernet Interface System provides the TCP attack protection and can be used for protecting the web service of the protection system. Web based automation systems designed using Raspberry PI and Arduino board have wired Ethernet interface. Such an automation system can be protected from SYN flood attack using the proposed Secured Ethernet Interface System. The Secured Ethernet Interface System is implemented as a SoC using NIOS processor and Triple-Speed Ethernet soft IP cores to protect the web server from SYN flood attack.

FPGA based Secured Ethernet Interface System is designed as SoC and logic utilization is only 8%. Hence, it should be possible to develop SoC that combines the features of embedded device used in automation system with the Secured Ethernet Interface System. The proposed Secured Ethernet Interface System is small in size and provides better transfer rate and improved speed. Further, this work can be modified to provide security features to the IOT devices that use Zigbee, 802.15.4, and so on. The secured Ethernet Interface System can be reprogrammed to protect the automation system from different types of DDoS attacks. The main limitation of this Secured Ethernet Interface System is at present limited to wired networks. But this system can be easily reprogrammed to be used in wireless networks.

## 6. Conclusion and Future Scope

Security of the network has become very significant on the Internet as malicious users generate attack so as to destruct the services on the server. In order to provide genuine client's access to the server and block the attack packets, the Secured Ethernet Interface System is designed. The* Secured Ethernet Interface System* designed using NIOS processor and Triple-Speed Ethernet is successfully implemented as a SoC. The logic utilization of* Secured Ethernet Interface System* is only 8%. Hence, it should be possible to develop SoC that combines the features of embedded device used in automation system with the Secured Ethernet Interface System.

The advantage of* Secured Ethernet Interface System* is identifying and blocking the SYN flood attack. Most of the SYN flood attack generated uses spoofed SYN attack and the proposed protection system detects and blocks the spoofed requests. The performance of the Secured Ethernet Interface System was studied by conducting various experiments. The experimental results show that the number of half-open connections set up on the protected server is decreased by 95%. The number of genuine client's requests served by the protected server increased during the attack as compared to when the server was not protected. The response time of the protected server is improved by a factor of 280 ms. The data transfer rate has improved to 2.25 Kbytes/sec for protected server, as compared to 1.05 Kb/sec under no protection. Hence the proposed FPGA based SYN flood attack protection system provides better security against the SYN flood attack.

The proposed design of* Secured Ethernet Interface System* uses sequential processing for protecting the server from SYN flood attack. Further, research can be carried out to design the hardware units like packet processing block, comparing the source IP address with the good registry block using bloom filter, and so on. These hardware independent blocks can be combined with the existing proposed FPGA design so that the FPGA based* Secured Ethernet Interface System* can process the tasks of protecting server in parallel, by which the performance of the* Secured Ethernet Interface System* can be further improved. Future work can be carried out using IPV6 since IPV4 is being substituted by IPV6. Further, this work can be modified to provide security to the IOT devices that use Zigbee, 802.15.4, and so on. The Secured Ethernet Interface System can be reprogrammed to protect the automation system from different types of DDoS attacks.

## Figures and Tables

**Table 1:** Transfer rate and time taken for test (500 genuine requests were sent in the presence of attack).

	Unprotected server	Protected server
Transfer rate (Kilobytes/sec)	1.05	2.25
Time taken for tests (seconds)	137.184	64.179
